# A new general integral transform for solving integral equations

**DOI:** 10.1016/j.jare.2020.08.016

**Published:** 2020-08-28

**Authors:** Hossein Jafari

**Affiliations:** a Department of Mathematics, University of Mazandaran, Babolsar, Iran; b Department of Mathematical Sciences, University of South Africa, UNISA0003, South Africa; c Department of Medical Research, China Medical University Hospital, China Medical University, Taichung 110122, Taiwan; d Department of Mathematics and Informatics, Azerbaijan University, Jeyhun Hajibeyli, 71, AZ1007, Baku, Azerbaijan

**Keywords:** Laplace transform, Fractional order integral equations, Integral equation, Ordinary differential equations, 31B10, 44A10, 26A33

## Abstract

**Introduction:**

Integral transforms are important to solve real problems. Appropriate choice of integral transforms helps to convert differential equations as well as integral equations into terms of an algebraic equation that can be solved easily.

During last two decades many integral transforms in the class of Laplace transform are introduced such as Sumudu, Elzaki, Natural, Aboodh, Pourreza, Mohand, G_transform, Sawi and Kamal transforms.

**Objectives:**

In this paper, we introduce a general integral transform in the class of Laplace transform. We study the properties of this transform. Then we compare it with few exiting integral transforms in the Laplace family such as Laplace, Sumudu, Elzaki and G\_transforms, Pourreza, Aboodh and etc.

**Methods:**

A new integral transform is introduced. Then some properties of this integral transform are discussed. This integral transform is used to solve this new transform is used for solving higher order initial value problems, integral equations and fractional order integral equation.

**Results:**

It is proved that those new transforms in the class of Laplace transform which are introduced during last few decades are a special case of this general transform. It is shown that there is no advantage between theses transforms unless for special problems.

**Conclusion:**

It has shown that this new integral transform covers those exiting transforms such as Laplace, Elzaki and Sumudu transforms for different value of *p*(*s*) and *q*(*s*). We used this new transform for solving ODE, integral equations and fractional integral equations. Also, we can introduce new integral transforms by using this new general integral transform.

## Highlights

• A new general integral transform which is covered all class of integral transform in the class of Laplace transform.
• We investigated the application of this new transform for solving ODE with constant and variable coefficient.
• This new transform can handle easily for fractional order integral equations and fractional order differential equations.
• We have discussed the advantage and disadvantage of other integral transformed which is defined during last 2 decades.
• We proved the related theorems for this new transform.

## Graphical abstract

**Figure:**
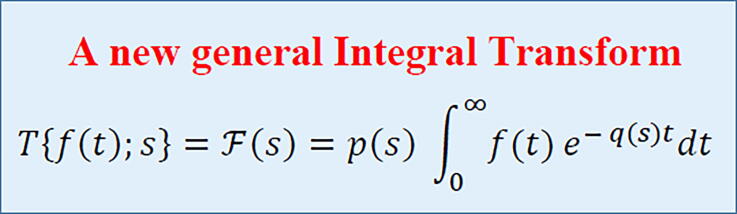

## Introduction

Integral transforms are precious for the simplification that they bring about, most often in dealing with differential equations subject to specific boundary conditions. Appropriate choice of integral transforms helps to convert differential equations and integral equations into terms of an algebraic equation that can be solved easily. The solution achieved is, of course, the transform of the solution of the original differential equation, and it is necessary to invert this transform to complete the operation [13], [23], [30], [37], [38]. Tables are available for the common transformations, those list many functions and their transforms.

During last two decades many integral transforms in the class of Laplace transform are introduced such as Sumudu, Elzaki, Natural, Aboodh, Pourreza, Mohand, G_transform, Sawi and Kamal transforms [1], [3], [6], [7], [14], [15], [18], [19], [20], [21], [34], [36]. In Table 1, we listed few of them with their definitions.

**Table 1:** Definition of some integral transforms

Laplace Transform	$L\{f(t)\}=\int_{0}^{\infty }f(t)e^{-\mathrm{st}}\mathrm{dt}$

Sumudu transform [36]	$S\{f(t)\}=\frac{1}{s}\int_{0}^{\infty }f(t)e^{-\frac{t}{s}}\mathrm{dt}$
Elzaki transform [15]	$E\{f(t)\}=s\int_{0}^{\infty }f(t)e^{-\frac{t}{s}}\mathrm{dt}$
Natural transform [21]	$N\{f(t)\}=R(s,u)=s\int_{0}^{\infty }f(\mathrm{ut})e^{-\mathrm{st}}\mathrm{dt}$
Aboodh transform [1]	$A\{f(t)\}=K(s)=\frac{1}{s}\int_{0}^{\infty }f(t)e^{-\mathrm{st}}\mathrm{dt}$
α-Integral Laplace Transform [27]	$L_{\alpha }\{f(t)\}=\int_{0}^{\infty }f(t)e^{-s^{\frac{1}{\alpha }}t}\mathrm{dt},\;\alpha \in R_{0}^{+}$
Pourreza transform [3], [5]	$\mathrm{HJ}\{f(t)\}=s\int_{0}^{\infty }f(t)e^{-s^{2}t}\mathrm{dt}$
Mohand transform [6]	$M\{f(t)\}=R(s)=s^{2}\int_{0}^{\infty }f(t)e^{-\mathrm{st}}\mathrm{dt}$
Sawi transform [7]	$\mathrm{Sa}\{f(t)\}=\frac{1}{s^{2}}\int_{0}^{\infty }f(t)e^{-\frac{t}{s}}\mathrm{dt}$
Kamal transform [18]	$K\{f(t)\}=G(s)=\int_{0}^{\infty }f(t)e^{-\frac{t}{s}}\mathrm{dt}$
G_transform [19], [20]	$G\{f(t)\}=F(s)=s^{\alpha }\int_{0}^{\infty }f(t)e^{-\frac{t}{s}}\mathrm{dt},\;\alpha$ is integer

These transforms have been used for solving different type of integral equations, ordinary differential equations (ODEs), partial differential equations (PDEs) and fractional differential equations (FDEs) as well as [5], [8], [10], [11], [12], [16], [23], [24], [25], [27], [32], [33], [38], [40]. Also the combination of these type transforms with other methods such as the Adomian decomposition and the homtopy perturbation methods has been used to solve various type of ODEs, PDEs and FDEs [2], [4], [17], [22], [28], [31], [39], [35].

In this work, a new integral transform is introduced and is used to obtain analytical solution of higher order ODES with constant and variable coefficient and integral equations. The paper is arranged as follows.

In Section ‘A new integral transform’, we introduce a general integral transform in the class of Laplace transform. In Section ‘Relation between the new transform and other transforms’, we compare the given integral transform with those existing integral transform in the class of Laplace transform. Then this integral transform is applied to ODEs and integral equations in Section ‘Solving IVP and Integral equations by new transform’. Finally, some conclusions are summarized in Section ‘Conclusion’.

## A new integral transform

In this section, we present a general integral transform which is cover most or even all type of integral transforms in the family of Laplace transform.

Definition 1 Let f(t) be a integrable function defined for t⩾0,p(s)≠0 and q(s) are positive real functions, we define the general integral transform T(s) of f(t) by the formula

(1) $$T\left\{f\left(t\right)\text{;}s\right\}=T\left(s\right)=p\left(s\right)\int_{0}^{\infty }f\left(t\right)e^{-q\left(s\right)t}\mathrm{dt},$$

provided the integral exists for some q(s).

Thus, we can obtain the integral transform of any general function. In Table 2, we provided new integral transform of some basic functions. It must be mentioned that the new integral transform (1) for those f(t) are not continuously differentiable contains terms with negative or fractional powers of q(s).

**Table 2:** Table of new integral transform.

Function	New integral transform
$f(t)=T^{-1}\{T(s)\}$	$T(s)=T\left\{f(t)\text{;}s\right\}$
1	$\frac{p(s)}{q(s)}$
*t*	$\frac{p(s)}{q{\left(s\right)}^{2}}$
$t^{\alpha }$	$\frac{\Gamma [\alpha +1]p(s)}{q{\left(s\right)}^{\alpha +1}},\;\alpha >0$
sint	$\frac{p(s)}{q{\left(s\right)}^{2}+1}$
sin(at)	$\frac{\mathrm{ap}(s)}{a^{2}+q{\left(s\right)}^{2}}$, if $q(s)>\left|I(a)\right|$
cost	$\frac{q(s)p(s)}{q{\left(s\right)}^{2}+1}$
$e^{t}$	$\frac{p(s)}{q(s)-1}$, q(s)>1,
tH(t-1)	$\frac{e^{-q(s)}(q(s)+1)p(s)}{q{\left(s\right)}^{2}}$
$f^{\prime }(t)$	q(s)T(s)-p(s)f(0)

Let for all t⩾0, the function f(t) is piecewise continuous and satisfies $|f(t)|⩽{\mathrm{Me}}^{\mathrm{kt}}$, then T(s) exists for all q(s)>k. Since

(2) $$\begin{array}{c} \|T\left\{f(t)\text{;}s\right\}\|=|p(s)\int_{0}^{\infty }f(t)e^{-q(s)t}\mathrm{dt}|⩽p(s)\int_{0}^{\infty }|f(t)|e^{-q(s)t}\mathrm{dt}\\ ⩽p(s)\int_{0}^{\infty }{\mathrm{Me}}^{\mathrm{kt}}e^{-q(s)t}\mathrm{dt}⩽\frac{p(s)M}{k-q(s)}, \end{array}$$

the statement is valid.

Theorem 1 *Let*f(t)*is differentiable and*p(s)*and*q(s)*are positive real functions, then*

(I) $T\left\{f^{\prime }(t)\text{;}s\right\}=q(s)T(s)-p(s)f(0)$,
(II) $T\left\{f^{''}\left(t\right)\text{;}s\right\}=q^{2}\left(s\right)T\left\{f\left(t\right)\text{;}s\right\}-q\left(s\right)p\left(s\right)f\left(0\right)-p\left(s\right)f^{'}\left(0\right)$,
(III) $T\left\{f^{\left(n\right)}\left(t\right)\text{;}s\right\}=q^{n}\left(s\right)T\left\{f\left(t\right)\text{;}s\right\}-p\left(s\right)\sum_{k=0}^{n-1}q^{n-1-k}\left(s\right)f^{\left(k\right)}\left(0\right)$.

Proof (I). In view of (1) we have

$$\begin{array}{c} T\left\{f^{'}\left(t\right)\text{;}s\right\}=p\left(s\right)\int_{0}^{\infty }f^{'}\left(t\right)e^{-q\left(s\right)t}\mathrm{dt}\\ =p\left(s\right)\left[e^{-q\left(s\right)t}f\left(t\right){\left(\right|}_{0}^{\infty }+q\left(s\right)\int_{0}^{\infty }f\left(t\right)e^{-q\left(s\right)t}\mathrm{dt}\right]\\ =q\left(s\right)T\left\{f\left(t\right)\text{;}s\right\}-p\left(s\right)f\left(0\right)\text{.} \end{array}$$

To proof (II), we assume $h(t)=f^{\prime }(t)$ so $f^{\prime \prime }(t)=h^{\prime }(t)$ now

(3) $$\begin{array}{c} T\left\{h^{'}\left(t\right)\text{;}s\right\}=p\left(s\right)\int_{0}^{\infty }h^{'}\left(t\right)e^{-q\left(s\right)t}\mathrm{dt}=q\left(s\right)T\left\{h\left(t\right)\text{;}s\right\}-p\left(s\right)h\left(0\right)\\ =q\left(s\right)T\left\{f^{'}\left(t\right)\text{;}s\right\}-p\left(s\right)f^{'}\left(0\right)\\ =q\left(s\right)\left[q\left(s\right)T\left\{f\left(t\right)\text{;}s\right\}-p\left(s\right)f\left(0\right)\right]-p\left(s\right)f^{'}\left(0\right)\\ =q^{2}\left(s\right)T\left\{f\left(t\right)\text{;}s\right\}-q\left(s\right)p\left(s\right)f\left(0\right)-p\left(s\right)f^{'}\left(0\right). \end{array}$$

By induction we can prove (III). □

Theorem 2 *(Convolution) Let*$f_{1}(t)$*and*$f_{2}(t)$*have new integral transform*$F_{1}(s)$*and*$F_{2}(s)$*. Then the new integral transform of the Convolution of*$f_{1}$*and*$f_{2}$*is*

(4) $$f_{1}*f_{2}=\int_{0}^{\infty }f_{1}(t)\;f_{2}(t-\tau )d\tau =\frac{1}{p(s)}F_{1}(s)\cdot F_{2}(s)\text{.}$$

Proof $$\begin{array}{c} T\left\{f_{1}*f_{2}\right\}=p\left(s\right)\int_{0}^{\infty }e^{-q\left(s\right)t}\int_{0}^{\infty }f_{1}\left(t\right)\;f_{2}\left(t-\tau \right)\mathrm{dt}\\ =p\left(s\right)\int_{0}^{\infty }f_{1}\left(\tau \right)d\tau \int_{0}^{\infty }e^{-q\left(s\right)t}f_{2}\left(t-\tau \right)\mathrm{dt}\\ =p\left(s\right)\int_{0}^{\infty }e^{-q\left(s\right)t}f_{1}\left(\tau \right)d\tau \int_{0}^{\infty }e^{-q\left(s\right)t}\;f_{2}\left(t\right)\mathrm{dt}=\frac{1}{p\left(s\right)}F_{1}\left(s\right)\cdot F_{2}\left(s\right)\text{.} \end{array}$$

 □

## Relation between the new transform and other transforms

In this section, we discuss about relation of the new integral transform (1) with the Laplace, α-Laplace, Sawi, Elzaki, Sumudu, Natural, Aboodh, Pourreza, Mohand, G_transform and Kamal transforms[15], [21], [36].

In view of (1) and definition of the above transforms which are listed in the Table 1, we have:

(i) If p(s)=1 and q(s)=s then this new transform gives the Laplace transform.
(ii) If p(s)=1 and $q(s)=s^{\frac{1}{\alpha }}$ then this new transform gives the α-Laplace transform.
(iii) If $p(s)=\frac{1}{s}$ and $q(s)=\frac{1}{s}$ then this new transform gives the Sumudu transform.
(iv) If $p(s)=\frac{1}{s}$ and q(s)=1 then this new transform gives the Aboodh transform.
(v) If p(s)=s and $q(s)=s^{2}$ then this new transform gives the Pourreza transform.
(vi) If p(s)=s and $q(s)=\frac{1}{s}$ then this new transform gives the Elzaki transform.
(vii) If p(s)=u and $q(s)=\frac{s}{u}$ then this new transform gives the Natural transform.
(viii) If $p(s)=s^{2}$ and q(s)=s then this new transform gives the Mohand transform.
(ix) If $p(s)=\frac{1}{s^{2}}$ and $q(s)=\frac{1}{s}$ then this new transform gives the Sawi transform.
(x) If p(s)=1 and $q(s)=\frac{1}{s}$ then this new transform gives the Kamal transform.
(xi) If $p(s)=s^{\alpha }$ and $q(s)=\frac{1}{s}$ then this new transform gives the G_transform.

It is easy to show that all of those integral transforms are a special cases of Eq. (1). In the next section, we can compare these transforms with classical Laplace transform. Also we get idea, that how can we choose one of these transforms and how can we define a suitable functions for p(s) and q(s) in the equation (1).

## Solving IVP and Integral equations by new transform

In this section, we apply this new integral transform for solving high order IVP with constant coefficient and variable coefficient as well as. Also we apply it to obtain exact solution of few type of integral equations and FDE.

### Solving IVP with constant coefficient

Consider the following IVP:

(5) $$y^{\left(n\right)}(t)+a_{1}\;y^{\left(n-1\right)}(t)+\cdots +a_{n}\;y(t)=g(x),$$

(6) $$y(0)=y_{0},\;y^{\prime }(0)=y_{1},\cdots ,\;y^{\left(n-1\right)}(0)=y_{n-1}\text{.}$$

Now we apply new integral transform to both side of equation (5). In view of Theorem 1, we have

(7) $$\begin{array}{c} T\left\{y^{\left(n\right)}\left(t\right)+a_{1}\;y^{\left(n-1\right)}\left(t\right)+\cdots +a_{n}\;y\left(t\right)\right\}=T\left\{g\left(x\right)\right\},\\ T\left\{y^{\left(n\right)}\left(t\right)\right\}+a_{1}\;T\left\{y^{\left(n-1\right)}\left(t\right)\right\}+\cdots +T\left\{a_{n}\;y\left(t\right)\right\}=T\left\{g\left(x\right)\right\}\text{.}\\ q^{n}\left(s\right)T\left(s\right)-p\left(s\right)\overset{n-1}{\underset{k=0}{\sum }}q^{n-1-k}\left(s\right)y^{\left(k\right)}\left(0\right)+a_{1}\left(q^{n-1}\left(s\right)T\left(s\right)\right)\\ \left(-p\left(s\right)\overset{n-2}{\underset{k=0}{\sum }}q^{n-2-k}\left(s\right)y^{\left(k\right)}\left(0\right)\right)\;+\cdots +a_{n}\;T\left(s\right)=G\left(s\right)\text{.} \end{array}$$

By substituting the initial conditions in the equation (7) we have

(8) h(s)T(s)=G(s)+Ψ(s),

where $h\left(s\right)=\left(q^{n}\left(s\right)+a_{1}q^{n-1}\left(s\right)+\cdots +a_{n}\right)\text{,}\;G\left(s\right)=T\left\{g\left(x\right)\right\}$ and

$\begin{array}{c} \Psi \left(s\right)=p\left(s\right)\left(\sum_{k=0}^{n-1}q^{n-1-k}\left(s\right)y_{k}+a_{1}\sum_{k=0}^{n-2}q^{n-2-k}\left(s\right)y_{k}+\cdots +y_{0}\right) \end{array}$. From (8), we find T(s) as

(9) $$T\left(s\right)=\frac{G\left(s\right)}{h\left(s\right)}+\frac{\Psi \left(s\right)}{h\left(s\right)}\text{.}$$

Finally, by apply the inverse transform $T^{-1}$ on both sides of the above equation, we obtain the exact solution:

(10) $$y\left(t\right)=T^{-1}\left\{\frac{G\left(s\right)}{h\left(s\right)}\right\}+T^{-1}\left\{\frac{\Psi \left(s\right)}{h\left(s\right)}\right\}\text{.}$$

Example 1 Consider the following third-order ODE

(11) $$y^{\prime \prime \prime }+y^{\prime \prime }-6y=0,$$

$$y(0)=1,\;y^{\prime }(0)=0,\;y^{\prime \prime }(0)=5\text{.}$$

By applying *T* on both side of (11) we have

(12) $$\begin{array}{c} T\left\{y^{'''}\left(t\right)+y^{''}\left(t\right)-6y\left(t\right)\right\}=T\left\{0\right\}\text{,}\\ T\left\{y^{'''}\left(t\right)\right\}+T\left\{y^{''}\left(t\right)\right\}-6T\left\{y\left(t\right)\right\}=0\text{,} \end{array}$$

$$q^{3}\left(s\right)T\left(s\right)-p\left(s\right)\left(q^{2}\left(s\right)y_{0}+q\left(s\right)y_{1}+y_{2}\right)+q^{2}\left(s\right)T\left(s\right)-p\left(s\right)\left(q\left(s\right)y_{0}+y_{1}\right)-6T\left(s\right)=0\text{,}$$

by replacing the initial conditions in above equation we have

$$\begin{array}{c} \left(q^{3}\left(s\right)+q^{2}\left(s\right)-6q\left(s\right)\right)T\left(s\right)=p\left(s\right)q^{2}\left(s\right)-p\left(s\right)+q\left(s\right)p\left(s\right)\text{.} \end{array}$$

So

(13) $$T\left(s\right)=\frac{p\left(s\right)q^{2}\left(s\right)-p\left(s\right)+q\left(s\right)p\left(s\right)}{q^{3}\left(s\right)+q^{2}\left(s\right)-6q\left(s\right)}=\frac{p\left(s\right)}{6q\left(s\right)}+\frac{1}{3}\frac{p\left(s\right)}{q\left(s\right)+3}+\frac{1}{2}\frac{p\left(s\right)}{q\left(s\right)-2}\text{.}$$

Now in view of Table 1, by applying $T^{-1}$ on both side of (18) we find the exact solution as

(14) $$\begin{array}{c} y\left(t\right)=\frac{1}{6}T^{-1}\left\{\frac{p\left(s\right)}{q\left(s\right)}\right\}+\frac{1}{3}T^{-1}\left\{\frac{p\left(s\right)}{q\left(s\right)+3}\right\}+\frac{1}{2}T^{-1}\left\{\frac{p\left(s\right)}{q\left(s\right)-2}\right\}\\ =\frac{1}{6}+\frac{1}{3}e^{-3t}+\frac{1}{2}e^{2t}\text{.} \end{array}$$

Example 2 Consider the following third-order ODE

(15) $$y^{\prime \prime \prime }(t)+2y^{\prime \prime }+2y^{\prime }(t)+3y(t)=\sin t+\cos t,$$

$$y\left(0\right)=y^{''}\left(0\right)=0,\;y^{'}\left(0\right)=1\text{.}$$

By applying *T* on both sides of (15) we have

(16) $$\begin{array}{c} T\left\{y^{'''}\left(t\right)\right\}+2T\left\{y^{''}\left(t\right)\right\}+2T\left\{y^{'}\left(t\right)\right\}+3T\left\{y\left(t\right)\right\}=T\left\{\sin t\right\}+T\left\{\cos t\right\}, \end{array}$$

$$\begin{array}{c} q^{3}\left(s\right)T\left(s\right)-p\left(s\right)\left(q^{2}\left(s\right)y_{0}+q\left(s\right)y_{1}+y_{2}\right)\\ +2\left[q^{2}\left(s\right)T\left(s\right)-p\left(s\right)\left(q\left(s\right)y_{0}+y_{1}\right)\right]\\ +2\left\{q\left(s\right)T\left(s\right)-p\left(s\right)y_{0}\right\}+3T\left(s\right)=\frac{p\left(s\right)}{q^{2}\left(s\right)+1}+\frac{q\left(s\right)p\left(s\right)}{q^{2}\left(s\right)+1}\text{,} \end{array}$$

by replacing the initial conditions in above equation, we have

$$\left[q^{3}(s)+2q^{2}(s)+2q(s)+3\right]T(s)=\frac{p(s)}{q^{2}(s)+1}+\frac{q(s)p(s)}{q^{2}(s)+1}+p(s)q(s)+2p(s),$$

by simplification, we get

(17) $$T(s)=\frac{p(s)}{q^{2}(s)+1}\text{.}$$

Now in view of Table 1, by applying $T^{-1}$ on both sides of (17) we find the exact solution as

(18) $$y(t)=T^{-1}\left\{\frac{p(s)}{q^{2}(s)+1}\right\}=\sin t\text{.}$$

Remark 1 All the above mentioned integral transforms for ODEs with constant coefficient give same solution.

Remark 2 In view of (10), if we choose the Laplace transform for ODEs with constant coefficient, the volume of calculation be minimum.

### Solving IVP with variable coefficient

Now consider the following type of IVP problems

(19) $$y^{\left(n\right)}\left(t\right)+a_{1}\left(t\right)\;y^{\left(n-1\right)}\left(t\right)+\cdots +a_{n}\left(t\right)\;y\left(t\right)=g\left(x\right)\text{,}$$

$$y(0)=y_{0},\;y^{\prime }(0)=y_{1},\cdots ,\;y^{\left(n-1\right)}(0)=y_{n-1}\text{.}$$

where $a_{i}(t)=b_{i}\;t^{m_{i}},\;b_{i}\in R\;\mathrm{and}\;m_{i}\in N$. It is clear that $m_{i}=0,1,2,\cdots ,n$ so we must find the integral transform of $T\left\{a_{i}(t)y^{\left(l\right)}(t)\right\}$. According to value of $a_{i}(t)$ and order of derivative $y^{\left(l\right)}\left(t\right),\;l=0,1,\cdots ,n-1$, we have different cases. In follow, we present few theorems which are useful to solve these type of IVP.

Theorem 3 *Let*p(s)*and*q(s)*are differentiable and*$q^{\prime }(s)\;\neq \;0$*, then*

(I) $T\left\{t\;f(t)\right\}=-\frac{p(s)}{q^{\prime }(s)}{\left(\frac{T(s)}{p(s)}\right)}^{\prime }$
(II) $T\left\{t^{2}\;f(t)\right\}={\left(-1\right)}^{2}\frac{p(s)}{q^{\prime }(s)}{\left(\frac{1}{q^{\prime }(s)}{\left(\frac{T(s)}{p(s)}\right)}^{\prime }\right)}^{\prime }$
(III) $T\left\{t^{n}\;f(t)\right\}={\left(-1\right)}^{n}\frac{p(s)}{q^{\prime }(s)}\underset{n-1\mathrm{times}}{\underset{︸}{\left(\frac{1}{q^{\prime }(s)}\left(\frac{1}{q^{\prime }(s)}\left(\cdots \left(\frac{1}{q^{\prime }(s)}\right)\right)\right)\right)}}{\left(\frac{T(s)}{p(s)}\right)}^{\prime }\underset{n-1\mathrm{times}}{\underset{︸}{{\left({\left(\right)}^{\prime }\cdots \right)}^{\prime }}}$

Proof (I). From definition we have

(20) $$T(s)=T\left\{f(t)\right\}=p(s)\int_{0}^{\infty }f(t)e^{-q(s)t}\mathrm{dt},$$

from the above equation we have

(21) $$\frac{T(s)}{p(s)}=\int_{0}^{\infty }f(t)e^{-q(s)t}\mathrm{dt},$$

now we take derivative on both sides of Eq. (21) respect to *s*, it leads

(22) $$\begin{array}{c} {\left(\frac{T\left(s\right)}{p\left(s\right)}\right)}^{'}=-q^{'}\left(s\right)\int_{0}^{\infty }t\;f\left(t\right)e^{-q\left(s\right)t}\mathrm{dt}=-q^{'}\left(s\right)\frac{T\left\{t\;f\left(t\right)\right\}}{p\left(s\right)}, \end{array}$$

after simplification we have

(23) $$T\left\{t\;f(t)\right\}=-\frac{p(s)}{q^{\prime }(s)}{\left(\frac{T(s)}{p(s)}\right)}^{\prime }\text{.}$$

(II). From (I) we have $p(s)\int_{0}^{\infty }t\;f(t)e^{-q(s)t}\mathrm{dt}=-\frac{p(s)}{q(s)}{\left(\frac{T(s)}{p(s)}\right)}^{\prime }$. Now by taking derivative form both sides of this equation we have $q^{\prime }(s)\int_{0}^{\infty }t^{2}\;f(t)e^{-q(s)t}\mathrm{dt}=-{\left(\frac{1}{q^{\prime }(s)}{\left(\frac{T(s)}{p(s)}\right)}^{\prime }\right)}^{\prime }$where $q^{'}(s)\int_{0}^{\infty }t^{2}\;f\left(t\right)e^{-q\left(s\right)t}\mathrm{dt}=\frac{T\left\{t^{2}\;f\left(t\right)\right\}}{p\left(s\right)}$, so substitute is there leads to

$$T\left\{t^{2}\;f(t)\right\}={\left(-1\right)}^{2}\frac{p(s)}{q^{\prime }(s)}{\left(\frac{1}{q^{\prime }(s)}{\left(\frac{T(s)}{p(s)}\right)}^{\prime }\right)}^{\prime }$$

Following the same procedure we can proof (III).  □

Theorem 4 *Let*p(s)*and*q(s)*are differentiable (*$q^{\prime }(s)\;\neq \;0$*), and*$f(t)\in C^{n}$*, then*

$$T\left\{t\;f^{\left(n\right)}(t)\right\}=-\frac{p(s)}{q^{\prime }(s)}\frac{d}{\mathrm{ds}}\left[\frac{1}{p(s)}T\left\{f^{\left(n\right)}(t)\right\}\right]\text{.}$$

Proof From definition (1), we have

(24) $$T\left\{f^{\left(n\right)}\left(t\right)\right\}=p\left(s\right)\int_{0}^{\infty }f^{\left(n\right)}\left(t\right)e^{-q\left(s\right)t}\mathrm{dt}\text{.}$$

By derivation of above equation respect to *s* we have

(25) $$\frac{d}{\mathrm{ds}}\left(\frac{1}{p\left(s\right)}T\left\{f^{\left(n\right)}\left(t\right)\right\}\right)=-q^{'}\left(s\right)\int_{0}^{\infty }t\;f^{\left(n\right)}\left(t\right)e^{-q\left(s\right)t}\mathrm{dt}=-q^{'}\left(s\right)\frac{T\left\{t\;f^{\left(n\right)}\left(t\right)\right\}}{p\left(s\right)}\text{.}$$

Thus

$$T\left\{t\;f^{\left(n\right)}(t)\right\}=-\frac{p(s)}{q^{\prime }(s)}\frac{d}{\mathrm{ds}}\left(\frac{1}{p(s)}T\left\{f^{\left(n\right)}(t)\right\}\right)\text{.}$$

 □

Theorem 5 *Let*p(s),q(s)*and*f(t)*are differentiable (*$q^{\prime }(s)\;\neq \;0$*), then*

$$\begin{array}{c} T\left\{t^{2}\;f^{\left(n\right)}\left(t\right)\right\}=\frac{p\left(s\right)}{q^{'}\left(s\right)}\frac{d}{\mathrm{ds}}\left[\frac{1}{q^{'}\left(s\right)}\left(\frac{d}{\mathrm{ds}}\left(\frac{1}{p\left(s\right)}T\left\{f^{\left(n\right)}\left(t\right)\right\}\right)\right)\right]\text{.} \end{array}$$

Proof From Theorem 4, we have

(26) $$T\left\{t\;f^{\left(n\right)}\left(t\right)\right\}=p\left(s\right)\int_{0}^{\infty }t\;f^{\left(n\right)}\left(t\right)\;e^{-q\left(s\right)t}\mathrm{dt}=-\frac{p\left(s\right)}{q^{'}\left(s\right)}\frac{d}{\mathrm{ds}}\left(\frac{1}{p\left(s\right)}T\left\{f^{\left(n\right)}\left(t\right)\right\}\right)\text{.}$$

By derivation of above equation respect to *s* we have

$$-\frac{d}{\mathrm{ds}}\left[\frac{1}{q^{'}\left(s\right)}\left(\frac{d}{\mathrm{ds}}\left(\frac{1}{p\left(s\right)}T\left\{f^{\left(n\right)}\left(t\right)\right\}\right)\right)\right]=-q^{'}\left(s\right)\int_{0}^{\infty }t^{2}\;f^{\left(n\right)}\left(t\right)e^{-q\left(s\right)t}\mathrm{dt}\;=-q^{'}\left(s\right)\frac{T\left\{t^{2}\;f^{\left(n\right)}\left(t\right)\right\}}{p\left(s\right)},$$

Thus

$$T\left\{t^{2}\;f^{\left(n\right)}\left(t\right)\right\}=\frac{p\left(s\right)}{q^{'}\left(s\right)}\frac{d}{\mathrm{ds}}\left[\frac{1}{q^{'}\left(s\right)}\left(\frac{d}{\mathrm{ds}}\left(\frac{1}{p\left(s\right)}T\left\{f^{\left(n\right)}\left(t\right)\right\}\right)\right)\right]\text{.}$$

 □

Theorem 6 *Let*p(s),q(s)*and*f(t)*are differentiable (*$q^{\prime }(s)\;\neq \;0$*), then*

(I) $T\left\{t^{n}\;f^{'}\left(t\right)\right\}={\left(-1\right)}^{n}\frac{p\left(s\right)}{q^{'}\left(s\right)}\frac{d}{\mathrm{ds}}\left[\underset{n\mathrm{times}}{\underset{︸}{\frac{1}{q^{'}\left(s\right)}\left(\frac{d}{\mathrm{ds}}\cdots \frac{1}{q^{'}\left(s\right)}\left(\frac{d}{\mathrm{ds}}\right)\right)}}\left(\frac{1}{p\left(s\right)}T\left\{f^{'}\left(t\right)\right\}\right)\left(\left(\right)\cdots \right)\right].$
(II) $T\left\{t^{n}\;f^{''}\left(t\right)\right\}={\left(-1\right)}^{n}\frac{p\left(s\right)}{q^{'}\left(s\right)}\frac{d}{\mathrm{ds}}\left[\underset{n\mathrm{times}}{\underset{︸}{\frac{1}{q^{'}}\left(\frac{d}{\mathrm{ds}}\cdots \frac{1}{q^{'}}\left(\frac{d}{\mathrm{ds}}\right)\right)}}\left(\frac{1}{p\left(s\right)}T\left\{f^{''}\left(t\right)\right\}\right)\left(\left(\right)\cdots \right)\right].$
(III) $T\left\{t^{n}\;f^{\left(m\right)}\left(t\right)\right\}={\left(-1\right)}^{n}\frac{p\left(s\right)}{q^{'}\left(s\right)}\frac{d}{\mathrm{ds}}\left[\underset{n\mathrm{times}}{\underset{︸}{\frac{1}{q^{'}}\left(\frac{d}{\mathrm{ds}}\cdots \frac{1}{q^{'}}\left(\frac{d}{\mathrm{ds}}\right)\right)}}\left(\frac{1}{p\left(s\right)}T\left\{f^{\left(m\right)}\left(t\right)\right\}\right)\left(\left(\right)\cdots \right)\right].$

Proof It is similar to Theorem 5. □

Remark 3 If q(s) be a constant, then we can not use that integral transform to solve ODE with variable coefficient.

Example 3 Consider the following second-order ODE with variable coefficient

(27) $$a\;t^{2}y^{''}\left(t\right)+b\;ty^{'}\left(t\right)+c\;y\left(t\right)=d\;t^{n},\;n\in \mathbb{N},$$

$$y(0)=y^{\prime }(0)=0\text{.}$$

where a,b,c and *d* are constants. We apply *T* on both side of the above equation:

(28) $$a\;T\left\{t^{2}y^{''}\left(t\right)\right\}+b\;T\left\{t\;y^{'}\right\}+c\;T\left\{y\left(t\right)\right\}=d\;T\left\{t^{n}\right\}\text{.}$$

Now in view of Theorem 3, Theorem 4, we have

(29) $$\begin{array}{c} a\frac{p\left(s\right)}{q^{'}\left(s\right)}\frac{d}{\mathrm{ds}}\left[\frac{1}{q^{'}\left(s\right)}\left(\frac{d}{\mathrm{ds}}\left(\frac{1}{p\left(s\right)}T\left\{y^{''}\left(t\right)\right\}\right)\right)\right]-b\frac{p\left(s\right)}{q^{'}\left(s\right)}\frac{d}{\mathrm{ds}}\left[\frac{1}{p\left(s\right)}T\left\{y^{'}\left(t\right)\right\}\right]\\ +c\;T\left\{y\left(t\right)\right\}=d\;T\left\{t^{n}\right\}\text{,}\\ a\frac{p\left(s\right)}{q^{'}\left(s\right)}\frac{d}{\mathrm{ds}}\left[\frac{1}{q^{'}\left(s\right)}\left(\frac{d}{\mathrm{ds}}\left(\frac{1}{p\left(s\right)}q^{2}\left(s\right)T\left(s\right)\right)\right)\right]-b\frac{p\left(s\right)}{q^{'}\left(s\right)}\frac{d}{\mathrm{ds}}\left[\frac{1}{p\left(s\right)}q\left(s\right)T\left(s\right)\right]\\ +c\;T\left(s\right)=n!d\frac{p\left(s\right)}{q^{n+1}\left(s\right)}\text{.} \end{array}$$

In the below, we listed the transformation of Eq. (29) for different integral transforms:

1. Laplace transform:  $\begin{array}{c} {\mathrm{as}}^{2}T^{''}\left(s\right)+s\left(4a-b\right)T^{'}\left(s\right)+T\left(s\right)\left(2a-b+c\right)-\frac{\mathrm{dn}!}{s^{n+1}}=0\text{.} \end{array}$
2. Pourreza transform:  $\begin{array}{c} {\mathrm{as}}^{2}T^{''}\left(s\right)+s\left(5a-2b\right)T^{'}\left(s\right)+T\left(s\right)\left(3a-2b+4c\right)-4\frac{\mathrm{dn}!}{s^{2n+1}}=0\text{.} \end{array}$
3. Elzaki transform:  $\begin{array}{c} {\mathrm{as}}^{2}T^{''}\left(s\right)+s\left(b-4a\right)T^{'}\left(s\right)+T\left(s\right)\left(6a-2b+c\right)-\mathrm{dn}!\;s^{n+1}=0\text{.} \end{array}$
4. Sawi transform:  $\begin{array}{c} {\mathrm{as}}^{2}T^{''}\left(s\right)+s\left(2a+b\right)T^{'}\left(s\right)+T\left(s\right)\left(b+c\right)-\mathrm{dn}!\;s^{n-1}=0\text{.} \end{array}$
5. Sumudu transform:  ${\mathrm{as}}^{2}T^{\prime \prime }(s)+\mathrm{bs}T^{\prime }(s)+cT(s)-\mathrm{dn}!\;s^{n}=0$.

Remark 4 It is clear that we still have another second order ODE. But for the Laplace, Pourreza and Elzaki transforms, we may have a simple second order ODE if coefficient of $T^{\prime }(s)$ and T(s) be zero.

For example, if in Eq. (28), we have a=2,b=5,c=1,d=24 and n=2, then we can apply the Pourreza transform as a best choice. It gives $T^{\prime \prime }(s)=\frac{96}{s^{7}}$. It can be easily solved. By solving this equation, we find $y(t)=\frac{8}{5}t^{2}$ which is the exact solution.

For another test example, Let a=1,b=4,c=2,d=12 and n=2 in Eq. (28), So the Elzaki transform is a best choice. It gives $T^{\prime \prime }(s)=24s^{2}$. By solving this equation, we find $y(t)=t^{2}$ which is the exact solution [16].

### Solving Integral equations

Example 4 Consider the following Volterra integral equation [20]

(30) $$y(t)=t+\int_{0}^{t}y(\tau )\sin(t-\tau )\mathrm{dt},$$

By applying this new transform on both sides of this equation and using Convolution Theorem we get

$$\begin{array}{c} Y\left(s\right)=T\left\{t\right\}+\frac{1}{p\left(s\right)}F\left(s\right)\cdot Y\left(s\right)⟶Y\left(s\right)=\frac{p\left(s\right)}{q{\left(s\right)}^{2}}+\frac{1}{p\left(s\right)}\frac{p\left(s\right)}{q{\left(s\right)}^{2}+1}Y\left(s\right)\text{,}\\ Y\left(s\right)=\frac{q{\left(s\right)}^{2}+1}{q{\left(s\right)}^{2}}\frac{p\left(s\right)}{q{\left(s\right)}^{2}}=\frac{p\left(s\right)q{\left(s\right)}^{2}}{q{\left(s\right)}^{4}}+\frac{p\left(s\right)}{q{\left(s\right)}^{4}}⟶y\left(t\right)=t+\frac{1}{6}t^{3}\text{.} \end{array}$$

Example 5 Consider the following Volterra integral equation [20]

(31) $$y\left(t\right)=1-\int_{0}^{x}\left(t-\tau \right)y\left(t\right)\mathrm{dt}\text{.}$$

By applying this new transform on both sides of this equation and using Convolution Theorem, we have

(32) $$\begin{array}{c} Y\left(s\right)=T\left\{1\right\}-\frac{1}{p\left(s\right)}F\left(s\right)\cdot Y\left(s\right)⟶Y\left(s\right)=\frac{p\left(s\right)}{q\left(s\right)}-\frac{1}{p\left(s\right)}\frac{p\left(s\right)}{q{\left(s\right)}^{2}}Y\left(s\right)\text{,}\\ Y\left(s\right)=\frac{q{\left(s\right)}^{2}}{q{\left(s\right)}^{2}+1}\frac{p\left(s\right)}{q\left(s\right)}=\frac{p\left(s\right)q\left(s\right)}{q{\left(s\right)}^{2}+1}⟶y\left(t\right)=\cos t\text{.} \end{array}$$

Example 6 Consider the following fractional integral equation

(33) $$y(t)=g(t)+I^{\alpha }y(t),\;\alpha \in R^{+},$$

where $I^{\alpha }$ is the well known Riemann–Liouville fractional integral operator [8], [9], [25], [26], [27], [29], [32], [33], [38]. It is defined by $I^{\alpha }y(t)=\frac{1}{\Gamma (\alpha )}\int_{0}^{t}{\left(t-\tau \right)}^{\alpha -1}y(\tau )d\tau$.

By substituting $\frac{1}{\Gamma (\alpha )}\int_{0}^{t}{\left(t-\tau \right)}^{\alpha -1}y(\tau )d\tau$ instead of $I^{\alpha }$ in Eq. (33) and apply Convolution Theorem 2, we have

(34) $$\begin{array}{c} Y\left(s\right)=T\left\{g\left(t\right)\right\}+\frac{1}{\Gamma \left(\alpha \right)p\left(s\right)}F\left(s\right)\cdot Y\left(s\right)\\ Y\left(s\right)=G\left(s\right)+\frac{1}{\Gamma \left(\alpha \right)p\left(s\right)}\frac{\Gamma \left(\alpha \right)p\left(s\right)}{q{\left(s\right)}^{\alpha }}Y\left(s\right),\\ Y\left(s\right)=\frac{1}{q{\left(s\right)}^{\alpha }-1}T\left\{g\left(t\right)\right\}⟶y\left(t\right)=T^{-1}\left\{\frac{1}{q{\left(s\right)}^{\alpha }-1}G\left(s\right)\right\}, \end{array}$$

where G(s) is $T\left\{g\left(t\right)\right\}$.

## Conclusion

In this paper, we introduce a general integral transform. After that we compare some integral transforms with this new integral transform. It has shown that the new integral transform cover those exiting transforms such as Laplace, Elzaki and Sumudu transforms for different value of p(s) and q(s). We used this new transform for solving ODE, integral equations and fractional integral equations. Few examples have been presented to illustrate the efficiency of this integral transform.

## Compliance with Ethics Requirements

*This article does not contain any studies with human or animal subjects*.

## Funding

Not applicable.

## Declaration of Competing Interest

The author declare that they have no conflict of interest.
